# Stability of magistral phage preparations before therapeutic application in patients with chronic rhinosinusitis, sepsis, pulmonary, and musculoskeletal infections

**DOI:** 10.1128/spectrum.02907-23

**Published:** 2023-10-11

**Authors:** Saartje Uyttebroek, Laura Bessems, Willem-Jan Metsemakers, Yves Debaveye, Laura Van Gerven, Lieven Dupont, Melissa Depypere, Jeroen Wagemans, Rob Lavigne, Maya Merabishvili, Jean-Paul Pirnay, David Devolder, Isabel Spriet, Jolien Onsea

**Affiliations:** 1 Department of Otorhinolaryngology, Head and Neck surgery, University Hospitals Leuven, Leuven, Belgium; 2 Department of Neurosciences, Experimental Otorhinolaryngology, Rhinology Research, KU Leuven, Leuven, Belgium; 3 Department of Trauma Surgery, University Hospitals Leuven, Leuven, Belgium; 4 Department of Development and Regeneration, KU Leuven, Leuven, Belgium; 5 Department of Intensive Care Medicine, University Hospitals Leuven, Leuven, Belgium; 6 Department of Cellular and Molecular Medicine, KU Leuven, Leuven, Belgium; 7 Department of Microbiology, Immunology and Transplantation, Allergy and Clinical Immunology Research Group, KU Leuven, Leuven, Belgium; 8 Department of Pneumology, University Hospitals Leuven, Leuven, Belgium; 9 Department of Chronic Diseases and Metabolism, Respiratory Diseases and Thoracic Surgery, KU Leuven, Leuven, Belgium; 10 Department of Laboratory Medicine, University Hospitals Leuven, Leuven, Belgium; 11 Department of Microbiology, Immunology and Transplantation, Laboratory of Clinical Bacteriology and Mycology, KU Leuven, Leuven, Belgium; 12 Department of Biosystems, Laboratory of Gene Technology, KU Leuven, Leuven, Belgium; 13 Laboratory for Molecular and Cellular Technology, Queen Astrid Military Hospital, Brussels, Belgium; 14 Pharmacy Department, University Hospitals Leuven, Leuven, Belgium; 15 Department of Pharmaceutical and Pharmacological Sciences, Clinical Pharmacology and Pharmacotherapy, KU Leuven, Leuven, Belgium; University of California San Diego, La Jolla, California, USA

**Keywords:** bacteriophages, phages, phage therapy, stability, formulation

## Abstract

Bacteriophages have (re)gained popularity as an adjunct antibacterial therapy. On top of the self-amplifying character of phages, their titer can be influenced by several factors which could lead to reduced titers after preparation and storage. The stability of phage preparations, used for the treatment of chronic rhinosinusitis (CRS), sepsis, pulmonary, and musculoskeletal infections, was investigated. *Staphylococcus aureus* phage ISP and *Pseudomonas aeruginosa* phages PNM, 14-1 and PT07 were evaluated. These phages were magistrally prepared by diluting active pharmaceutical products and storing them in different polypropylene/polycarbonate syringes at 4°C. Titers were monitored for 7–21 days using the double-agar overlay method. For 7 days, titers remained stable. Afterwards, the phage titer reduced for all phages with an average reduction of 0.78 log (*P* < 0.0001) plaque-forming units (PFUs)/mL after 14 days and 1 log PFU/mL after 21 days (*P* < 0.0001). PT07 titers dropped below the predefined threshold after 2 days in polycarbonate syringes. In addition, the stability after irrigation using a nasal douche (CRS) and infusion through a catheter (sepsis) was assessed. No changes were observed after using these devices with a mean change in titer of −0.07 log PFU/mL (*P* = 0.9062) and −0.03 log PFU/mL (*P* = 0.7350), respectively. In conclusion, a decreased titer after storage of a phage solution can lead to administration of subtherapeutic concentrations. In our experiments, we demonstrated that liquid phage solutions can be stored in syringes at 4°C for 5–7 days. PT07 appeared to be less stable, especially in polycarbonate syringes. Irrigation using a nasal douche or infusion through a catheter does not impact phage titer.

**IMPORTANCE:**

As antimicrobial resistance becomes more prevalent, the application of (bacterio)phage therapy as an alternative treatment for difficult-to-treat infections is (re)gaining popularity. Over the past decade, numerous promising case reports and series have been published demonstrating the therapeutic potential of phage therapy. However, important questions remain regarding the optimal treatment protocol and, unlike for medicinal products, there are currently no predefined quality standards for the stability of phage preparations. Phage titers can be influenced by several factors which could lead to reduced titers after preparation and storage and, ultimately, subtherapeutic applications. Determining the stability of different phages in different recipients according to the route of administration is therefore one of the first important steps in establishing a standardized protocol for phage therapy.

## INTRODUCTION

**Fig 1:**
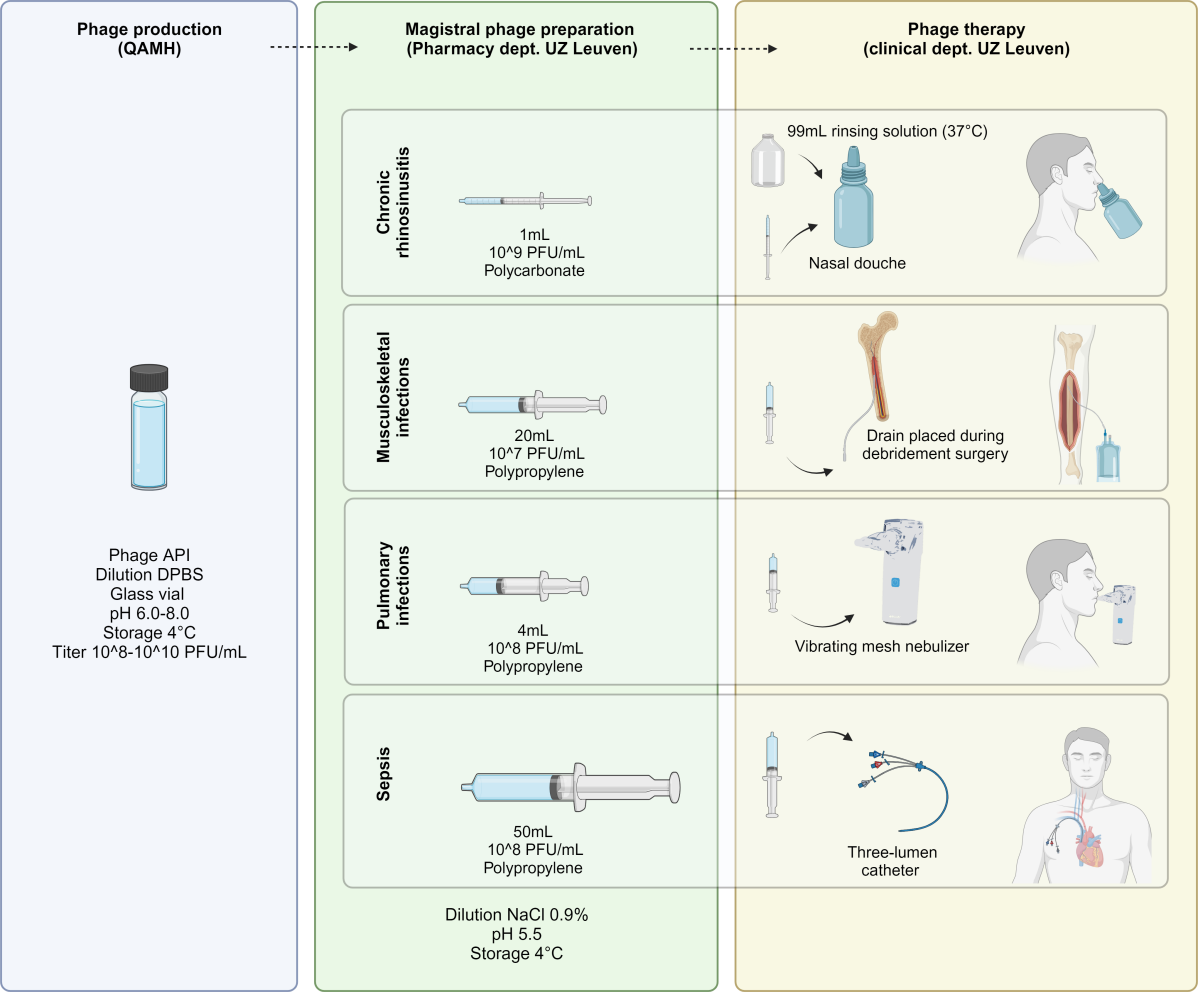
Phage therapy flow in the PHAGEFORCE project: from magistral phage preparation to standardized treatment protocols. Figure created with Biorender.com. QAMH: Queen Astrid Military Hospital, UZ Leuven: University Hospitals Leuven, API: active pharmaceutical ingredient, DPBS: Dulbecco’s phosphate-buffered saline, PFU: plaque-forming units.

In 2018, a framework for the use of (bacterio)phages as magistral preparations was approved in Belgium (1). A magistral preparation is defined as “any medical product prepared in a pharmacy in accordance with a medical prescription for an individual patient” (Article 3 of Directive 2001/83 and Article 6 quarter, §3 of the Law of 25 March 1964). This framework implies that phages can be processed as active pharmaceutical ingredients (APIs) and delivered as magistral preparations, after prescription by a medical doctor, to a specific patient. A monograph defines the manufacturing process, the characteristics and quality standards of phage APIs for human applications (1). A Belgian Approved Laboratory (Sciensano) is assigned to perform quality assessment. Considering this national strategy, a task force referred to as the Coordination group for Bacteriophage therapy Leuven (CBL) and consisting of various specialists in the field, was set up in our center in collaboration with the Laboratory for Molecular and Cellular Technology at the Queen Astrid Military Hospital (QAMH) (2). The CBL selects patients eligible for phage therapy (PT) and uses a standardized treatment and follow-up protocol. To date, patients with difficult-to-treat chronic rhinosinusitis (CRS), musculoskeletal infections (MSI), pulmonary infections in patients with cystic fibrosis or bronchiectasis (CF/Bx), and sepsis, are eligible for PT at our center (2). The QAMH provides purified APIs, produced according to the monograph and approved by Sciensano. Subsequently, magistral phage solutions are prepared at the Pharmacy department of University Hospitals Leuven (Fig. 1). Currently approved phages for phage therapy in Belgium include ISP, an *S. aureus* directed phage which covers ~73% of clinical isolates (3), and three *P. aeruginosa* directed phages, including two myoviruses 14-1 and PT07 and one podovirus, PNM. These phages are most widely used to treat patients with difficult-to-treat bacterial infections in our country.

The stability of phage solutions can be influenced by several factors including phage morphology, formulation (e.g., liquid, dry powder, and ointment) (4, 5), temperature, light exposure, pH, and buffer (6, 7). Furthermore, little is known about the impact of handling or packaging materials (e.g., glass vials vs polypropylene syringes) (8). An example underlining the importance of stability testing is the Phagoburn trial where patients were treated subtherapeutically due to storage issues leading to a decreased titer (9). With this study, we aimed to determine the stability of magistral ISP, PNM, 14-1, and PT07 phage solutions, prepared according to standardized protocols and stored under the conditions used for the treatment of CRS, MSI, CF/Bx, and sepsis in our center as part of the PHAGEFORCE project (2). The impact of storage in polycarbonate/polypropylene syringes and the use of a device (nasal douche for CRS and three-lumen catheter for sepsis) prior to treatment was assessed to ensure the deposition of sufficient numbers of active phages at the location of infection.

## RESULTS

### Stability of magistral phage preparations in polypropylene/polycarbonate syringes

#### Chronic rhinosinusitis

**Fig 2:**
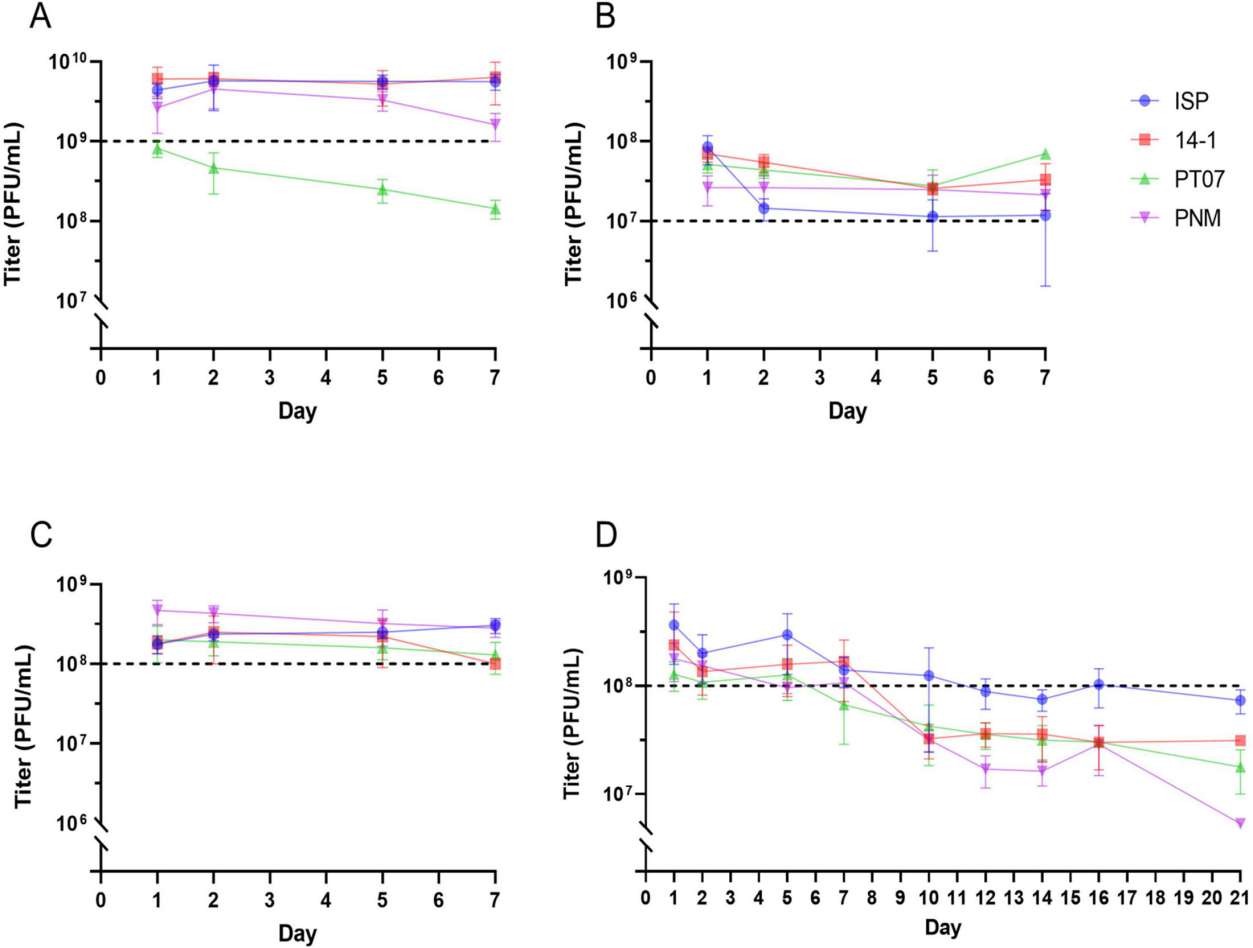
Repeated phage titers with mean and standard deviation for CRS (**A**), MSI (**B**), sepsis (**C**), and CF/Bx (**D**). PFU: plaque-forming units.

To determine the stability of 1 mL phage solutions for intranasal applications in patients with difficult-to-treat CRS, the phage titer was measured repeatedly for 7 days (Fig. 2A). A solution with a starting titer of 4.4 × 10^9^ (SD ± 9.5×10^8^), 6.1 × 10^9^ (SD ± 2.4 × 10^9^), 8.4 × 10^9^ (SD ± 1.9 × 10^8^) and 2.7 × 10^9^ (SD ± 1.4 × 10^9^) plaque-forming units (PFU)/mL was prepared for ISP, 14-1, PT07, and PNM, respectively. After 7 days, no significant reduction in ISP (mean change = 0.107 log PFU/mL, *P* = 0.1335), 14-1 (mean change = 0.011 log PFU/mL, *P* = 0.9455), and PNM (mean change = −0.190 log PFU/mL, *P* = 0.2089) titers was observed (Fig. 2A; Table 1). By contrast, PT07 titers dropped below the predefined effective therapeutic threshold (ETT) of 1 × 10^9^ PFU/mL after 2 days with a mean reduction of 0.760 log PFU/mL after 7 days (*P* < 0.0001).

**TABLE 1:** Stability of phage preparations for chronic rhinosinusitis, musculoskeletal infections, and sepsis

Indication (therapeutic threshold) Syringe Diluent	Phage	Day 1 vs day 5			Day 1 vs day 7		
		Mean Δ (log PFU/mL)	*P* value	95% CI	Mean Δ (log PFU/mL)	*P* value	95% CI
**Chronic rhinosinusitis** (1 × 10^9^ PFU/mL) 1 mL polycarbonate NaCl 0.9%	ISP	0.111	0.2641	[−0.32 to 0.09]	0.107	0.1335	[−0.25 to 0.04]
	14-1	0.003	0.8438	[−0.48 to 0.12]	0.011	0.9455	[−0.44 to 0.42]
	PT07	0.528	<0.0001^*a*^	[−0.64 to −0.42]	0.760	<0.0001^***a***^	[−0.92 to −0.60]
	PNM	0.088	0.4375	[−0.22 to 0.48]	0.190	0.2089	[−0.53 to 0.15]
	Phages combined	0.070	0.0595	[−0.50 to 0.10]	0.136	0.0852	[−0.40 to 0.05]
**Musculoskeletal infections** (1 × 10^7^ PFU/mL) 20 mL polypropylene NaCl 0.9%	ISP	0.786	0.0039^*a*^	[−1.16 to −0.41]	0.818	0.0043^*a*^	[−1.22 to 0.42]
	14-1	0.431	<0.0001^*a*^	[−0.53 to −0.33]	0.251	0.0765	[−0.54 to 0.04]
	PT07	0.197	0.056	[−0.01 to 0.40]	0.164	0.6318	[−0.77 to 0.44]
	PNM	0.465	0.0074^*a*^	[−0.75 to −0.18]	0.543	0.0012^*a*^	[−0.76 to −0.32]
	Phages combined	0.240	0.0101^*a*^	[−0.43 to −0.05]	0.360	<0.0001^*a*^	[−0.51 to −0.21]
**Sepsis** (1 × 10^8^ PFU/mL) 50 mL polypropylene NaCl 0.9%	ISP	0.157	0.0051^*a*^	[−0.24 to −0.07]	0.241	0.0210^*a*^	[−0.43 to −0.05]
	14-1	0.055	0.9127	[−0.46 to 0.57]	0.233	0.0356^*a*^	[−0.44 to −0.02]
	PT07	0.062	0.8321	[−0.33 to 0.45]	0.167	0.1019	[−0.05 to 0.38]
	PNM	0.180	0.0639	[−0.02 to 0.37]	0.210	0.0070^*a*^	[0.083 to 0.34]
	Phages combined	0.008	0.9903	[−0.15 to 0.16]	0.065	0.5244	[−0.09 to 0.22]

^*a*^ *P* value < 0.05 is considered statistically significant, CI = confidence interval, Δ = change. PFU: plaque-forming units.

#### Musculoskeletal infections

For the treatment of MSI, phage solutions are prepared in a 20 mL polypropylene syringe. The phage titer was measured repeatedly for 7 days (Fig. 2B). Solutions were prepared with baseline titers of 8.4 × 10^7^ (SD ± 3.4 × 10^7^) for ISP, 7.0 × 10^7^ (SD ± 1.5 × 10^7^) for 14-1, 5.1 × 10^7^ (SD ± 1.1 × 10^7^) for PT07, and 2.6 × 10^7^ (SD ± 1.1 × 10^7^) PFU/mL for PNM. Over 7 days, the titers remained stable for PT07 (mean change = −0.164 log PFU/mL, *P* = 0.6318) and 14-1 (mean change = −0.251 log PFU/mL, *P* = 0.0765), whereas a significant reduction was observed after 7 days for ISP (mean change = −0.818 log PFU/mL, *P* = 0.0043) and PNM (mean change = −0.543 log PFU/mL, *P* = 0.0012). All phage titers remained above the ETT of 1 × 10^7^ log PFU/mL (Fig. 2B; Table 1).

#### Pulmonary infections

The titer of phage solutions stored in a 5 mL polypropylene syringe used for nebulization in pulmonary infections was assessed for 21 days (Fig. 2D). Magistral preparations with baseline titers of 3.6 × 10^8^ (SD ± 2.0 × 10^8^), 2.4 × 10^8^ (SD ± 2.4 × 10^8^), 1.3 × 10^8^ (SD ± 3.9 × 10^7^), and 1.8 × 10^8^ (SD ± 6.9 × 10^7^) PFU/mL were prepared for ISP, 14-1, PT07, and PNM, respectively. The first 7 days, the titer remained stable, except for PT07 (mean change = −0.525 log PFU/mL, *P* = 0.0103). After 7 days, the titer of all phages dropped below the ETT of 1 × 10^8^ PFU/mL and significantly lower titers were observed compared to baseline for ISP (mean change = −0.638 log PFU/mL, *P* = 0.0208), PT07 (mean change = −0.618 log PFU/mL), and PNM (mean change = −1.029 log PFU/mL, *P* = 0.0064). After 21 days, a mean reduction of −0.646 (*P* = 0.6301*),* –0.748 (*P* = 0.4267), –0.863 (*P* = 0.1732), and −1.5 log PFU/mL (*P* = 0.0847) was observed for ISP, 14-1, PT07, and PNM, respectively (Fig. 2D, Table 2).

**TABLE 2:** Stability of phage preparations for pulmonary infections in cystic fibrosis and bronchiectasis patients

Indication (therapeutic threshold) Syringe Diluent	Phage	Day 1 vs day 7			Day 1 vs day 14			Day 1 vs day 21		
		Mean Δ (log PFU/mL)	*P* value	95% CI	Mean Δ (log PFU/mL)	*P* value	95% CI	Mean Δ (log PFU/mL)	*P* value	95% CI
**Pulmonary infections** (1 × 10^8^ PFU/mL) 4 mL polypropylene NaCl 0.9%	ISP	0.373	0.2297	[−0.99 to 0.24]	0.638	0.0208^*a*^	[−1.15 to −0.12]	0.646	0.6301	[−12.46 to 11.17]
	14-1	0.073	0.9729	[−1.01 to 0.87]	0.725	0.0814	[−1.60 to 0.15]	0.748	0.4267	[−7.72 to 6.23]
	PT07	0.525	0.0103^*a*^	[−0.86 to 0.020]	0.618	0.0034^*a*^	[−0.91 to −0.33]	0.863	0.1732	[−3.89 to 2.17]
	PNM	0.200	0.2593	[−0.71 to 0.31]	1.029	0.0064^*a*^	[−1.52 to −0.54]	1.500	0.0847	[−4.05 to 1.05]
	Phages combined	0.367	0.0024^*a*^	[−0.62 to −0.11]	0.779	<0.0001^*a*^	[−1.02 to −0.54]	0.996	<0.0001^*a*^	[−1.31 to −0.68]

^*a*^ *P* value < 0.05 is considered statistically significant, CI = confidence interval, Δ = change. PFU: plaque-forming units.

#### Sepsis

For sepsis, the stability of phage solutions prepared in 50 mL polypropylene syringes was assessed during 7 days (Fig. 2C). Phage solutions with starting titers of 1.8 × 10^8^ (SD ± 4.3 × 10^7^) for ISP, 1.8 × 10^8^ (SD ± 4.6 × 10^7^) for 14-1, 2.0 × 10^8^ (SD ± 9.7 × 10^7^) for PT07, and 4.7 × 10^8^ PFU/mL (SD ± 1.6 × 10^7^) for PNM were prepared. After 7 days, a significant reduction in titer of 14-1 (mean change = −0.233 log PFU/mL, *P* = 0.241) and PNM (mean change = −0.210 log PFU/mL, *P* = 0.0070) was observed, but all titers remained above the ETT of 1 × 10^8^ PFU/mL during the entire experiment (Fig. 2C; Table 1).

### Mechanical tests for CRS and sepsis

#### Nasal irrigation using a nasal douche

**Fig 3:**
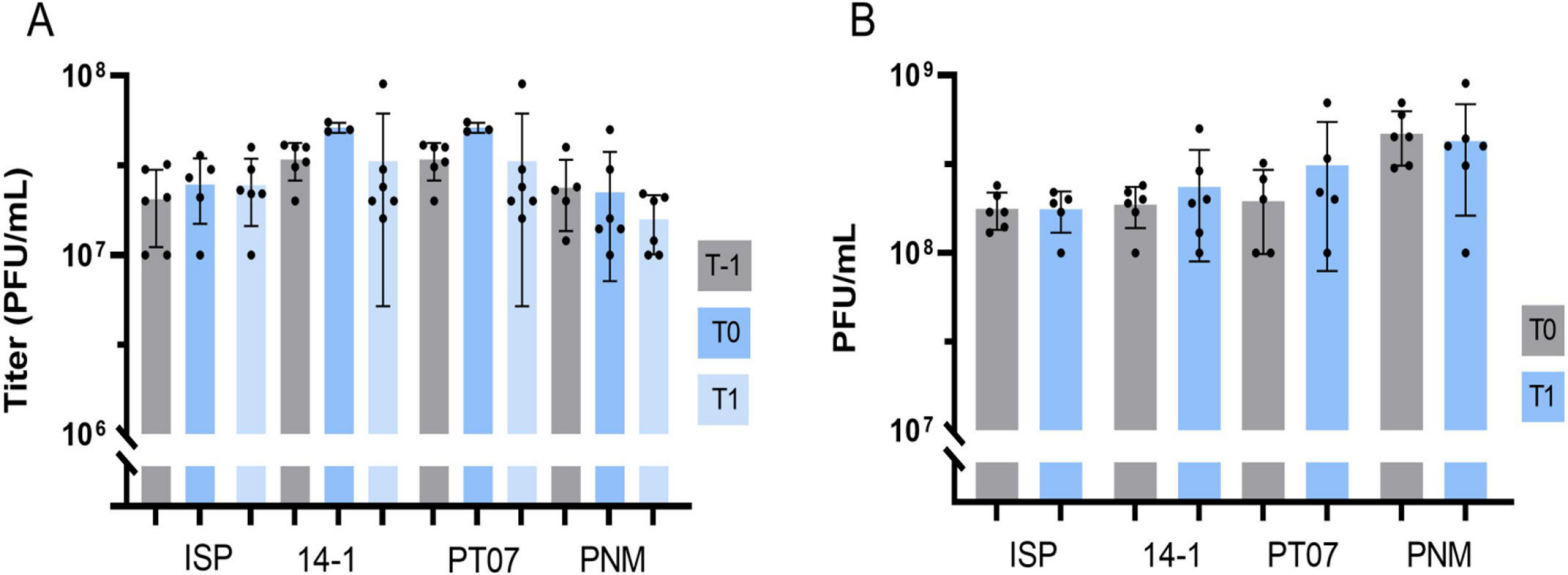
(A) Mechanical test for CRS with baseline titer (T-1), titer after irrigation (T0) and titer after 1 h at room temperature. (B) Mechanical test for sepsis with baseline titer (T0) and titer after infusion through a three-lumen catheter (T1). PFU: plaque-forming units.

Mechanical tests were carried out for CRS to determine the impact of applying positive pressure to a phage solution by squeezing a nasal douche containing 100 mL of a phage solution with a concentration of 10^7^ PFU/mL (Fig. 3A). Titers of all phages remained stable after the application of positive pressure immediately after dilution (mean change = −0.072 log PFU/mL, *P* = 0.9062) and after storage at room temperature for 1 h (mean change = −0.104 log PFU/mL, *P* = 0.3490) (Fig. 3A).

#### Infusion through three-lumen catheter

For sepsis, the stability of phage solutions was assessed after (bolus) infusion of 50 mL through a three-lumen catheter for 5 min. After infusion, the titers of ISP (mean change = 0.043 PFU/mL, *P* = 0.9350), 14-1 (mean change = 0.079 PFU/mL, *P* = 0.9810), PT07 (mean change = 0.1598 PFU/mL, *P* = 0.7034), and PNM (mean change = −0.10 log PFU/mL, *P* = 0.7979) remained stable and above the ETT (Fig. 3B).

### Tween 20 adsorption experiment

**Fig 4:**
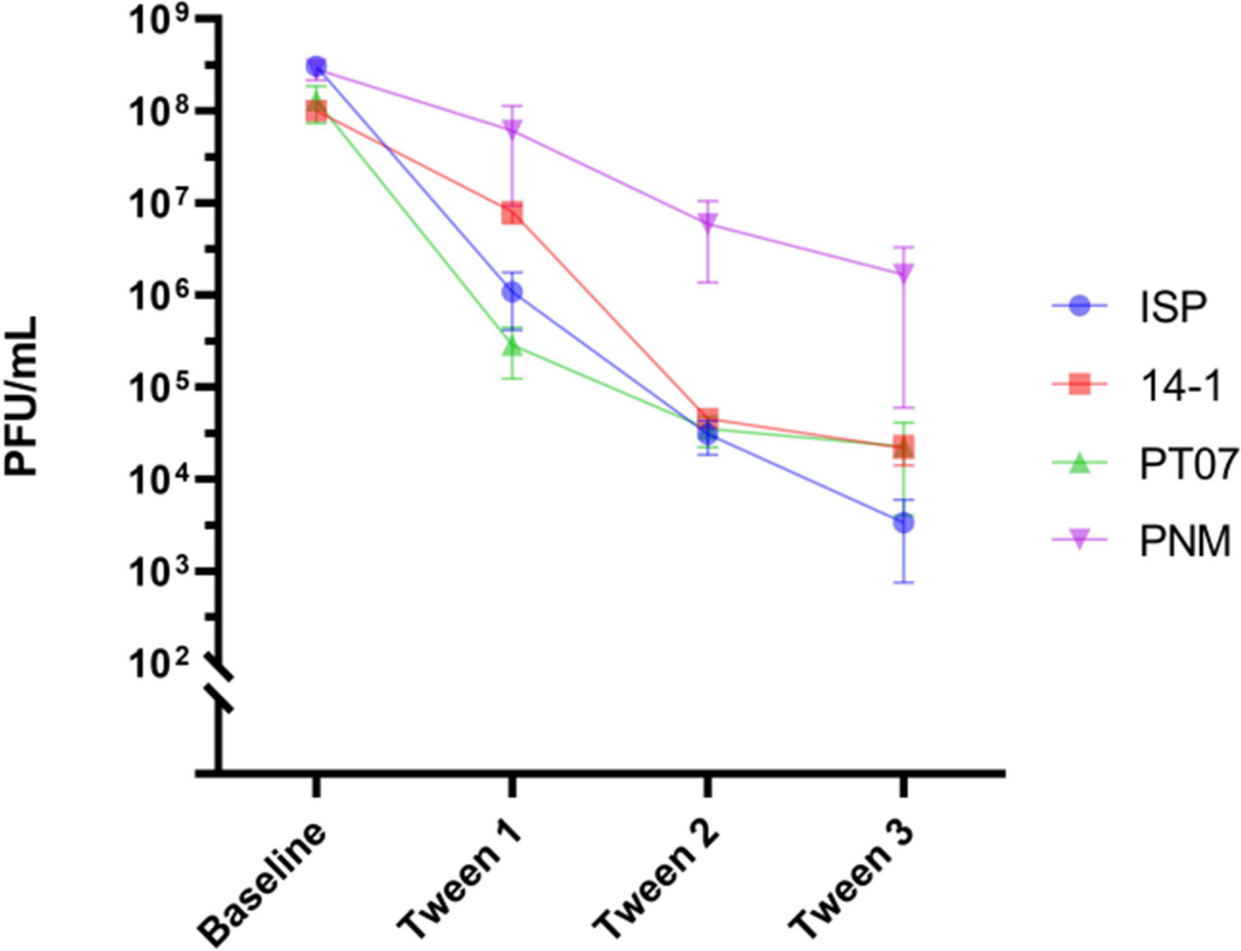
Number of phages (PFU/mL) in the initial phage solution (baseline) and attached to the 50 mL polypropylene syringe after three rounds of washing with Tween 20. PFU: plaque-forming units.

The number of phages attached to the inner surface of the 50 mL polypropylene syringe was calculated at baseline and after three rounds of washing with surfactant Tween 20. After three rounds of washing, the titer of the final phage solutions retrieved from the syringes was 3.3 × 10^3^ PFU/mL for ISP, 2.17 × 10^4^ PFU/mL for 14-1, 2.25 × 10^4^ PFU/mL for PT07, and 1.67 × 10^5^ PFU/mL for PNM, respectively. A total number of 5.58 × 10^7^, 4.3 × 10^6^, 1.48 × 10^9^, and 1.61 × 10^7^ active phage particles (PFU) of ISP, 14-1, PNM, and PT07, respectively, was retrieved from the polypropylene, indicating that a high number of phages attach to the plastic (Fig. 4).

## DISCUSSION

Bacteriophages are a promising adjunct treatment in the battle against difficult-to-treat infections. Despite the high efficacy of phages *in vitro*, qualitative pharmaceutical and clinical data are scarce (10). The knowledge of phage stability is one of the cornerstones to effectively treat patients, and to avoid subtherapeutic applications.

The stability of magistral phage preparations can be influenced by several factors, including formulation, buffer, temperature, ion composition, pH, and light exposure (7). Several techniques have been described to enhance stability for long-term storage after the production process, including encapsulation and lyophilization (freeze-drying) (4, 11 – 13). By contrast, there is little data available on the stability of phage solutions after dilution at the hospital pharmacy and after storage at the patients’ home. Furthermore, preferred storage recipients have not been specifically defined.

To optimize our protocol in the context of the PHAGEFORCE project (2), experiments to determine the stability of magistral phage solutions were set up. ETTs were defined after consensus by the CBL to ensure sufficiently high phage levels before therapeutic application, and to improve standardization of treatment protocols in our center. Our data show that titers of ISP, PNM, and 14-1 are relatively stable for the first 5–7 days and remain above the predefined ETT. However, PT07 titers were significantly reduced below the ETT after 2 days in polycarbonate syringes (CRS) and after 7 days in polypropylene syringes. After 7 days, the titer decreased for all phages (CF/Bx) with a mean reduction ranging from −0.65 to −1.5 log PFU/mL after 21 days.

Several factors contribute to the instability of these solutions, including their relatively low titers, when compared to titers used for storage of APIs (10^10^–10^11^ PFU/mL). Duyvejock et al. demonstrated that storage of PNM, ISP, and 14-1 phage at 10^7^ PFU/mL resulted in a major decrease in phage titer after 14 days, independent of phage morphology or storage solutions (6). Furthermore, in our center, NaCl 0.9% was used to dilute APIs instead of specific buffers due to its safety profile when administered in patients. Previous literature has shown that NaCl 0.9% is inferior to other solutions, including DPBS, especially at lower titers (6). Other factors include phage morphology, driven by their structure and electrostatic properties, and adsorption of phages to polymeric materials. The latter is supported by Richter et al., who found a variation of up to five logs due to adsorption to plastic. The adsorption was objectified by scanning electron microscopy, atomic force microscopy, and applying the surfactant Tween 20 (8). In our experiments, the adsorption of phages to the inner wall of a 50 mL polypropylene syringe was determined by repeatedly rinsing with Tween 20. In total, 10^6^–10^9^ active phages were retrieved after three rounds, indicating that a large portion of phages attach to the polymer surface of the syringe.

Finally, our mechanical tests show that phages can be safely used in a nasal irrigation system by applying positive pressure, and administered intravenously (in bolus over 5 min) through a catheter.

Based on these findings, it is clear that stability testing is of key importance before application of phage preparations in patients, and this should be optimized for each specific phage, storage solution, recipient, and application method. Ideally, these measurements are predefined by regulatory agencies, as is the case for classic medicinal products. This could be arranged on a national level, but—to promote standardization—preferably on a European level, whereby scientific organizations such as the recently formed ESCMID Study Group for Non-traditional Antibacterial Therapy (ESGNTA) could play an important role (14). We suggest storing preparations in syringes for no longer than 5–7 days at 4°C. Afterwards, a new batch should be prepared to ensure sufficiently high titers. In the next step, the possible interaction of phages within a cocktail should be evaluated. Within a cocktail, phage-phage interactions may occur, leading to synergism or antagonism, and subsequently different infection patterns than expected based on the host range of the phages separately (6).

The main limitation of this study is that only phages that are available as APIs in Belgium, with predefined titers and stored in specific storage recipients were tested. No comparison was made between several phage concentrations or storage recipients and no evaluation of phage-phage interactions was performed. We also evaluated the phage stability of solutions with a fixed pH of 5.5 that is used in our clinical setting, and we did not test a range of pH values. Future studies should investigate these factors contributing to (in)stability of phage preparations. Additionally, the adhesion of phage particles to polymer materials (including syringes and catheters) could be further evaluated, for instance using scanning electron microscopy, as indicated by Richter et al. (8).

## MATERIALS AND METHODS

### Treatment protocols

The treatment protocol, including application method, dosage, titer, and volume, for each indication was carefully designed after multidisciplinary consensus by the CBL, in the context of the PHAGEFORCE project (2).

At our center, phage therapy is applied locally for each treatment indication, except for sepsis (Fig. 1). For CRS, phages are applied intranasally using a nasal douche (Nasofree). The patient is asked to combine 1 mL of a magistrally prepared phage solution with 99 mL of sodium bicarbonate enriched rinsing salt, in the nasal douche. Afterwards, the patient is asked to rinse the nose by squeezing the bottle, three times a day. In the case of MSI, phages are applied through a drainage system that is placed during surgery, after debridement. Three times a day, the drainage system is rinsed with 20 mL of a phage solution. For CF/Bx, the patient is asked to add 4 mL of a phage solution in a mesh nebulizer and nebulize four times a day. Patients with severe sepsis are treated intravenously using a three-lumen catheter. About 50 mL is then administered over 5 min.

The CBL came to a consensus that a phage solution should contain 10^7^ PFU/mL active phages before application in patients with CRS and MSI. For CF/Bx and sepsis, a titer of 10^8^ PFU/mL was chosen, to anticipate for a reduced titer after inhalation through a nebulizer (CF/Bx), and intravenous distribution (sepsis). These titers were defined as ETT. As no literature is available on the optimal dosage, the titer was based on years of experience by the Eliava Institute and the general rule that phage preparations should contain at least 10^6^–10^7^ PFU/mL phage particles (15).

The choice of phages, including one *S. aureus* (ISP) and three *P. aeruginosa* (14-1, PNM, and PT07) phages, was made based on the predominance of *S. aureus* and *P. aeruginosa* infections in the indications that are treated in our center (16, 17). Moreover, these phages are approved by Sciensano to be used as APIs in magistral preparations and are most widely used to treat patients with difficult-to-treat infections in our country. The characteristics of these phages are described in Table 3.

**TABLE 3:** Phage characteristics

Phage	Phage classification (family, genus)	Phage morphology	Propagation strain	Genome accession #	Genome size (kb)	Phage receptor	Host range (% of clinical isolates)	Refs
ISP	*Herelleviridae*, *Kayvirus*	Myovirus	*S. aureus* ATCC 6538	NC_047720 NC_047720	138.3	WTA^*a*^	73%^*b*^	(3, 18)
14-1	*Caudoviricetes*, *Pbunavirus*	Myovirus	*P. aeruginosa* CN573	NC_011703	66.1	LPS	25%^*c*^	(19)
PNM	*Autographiviridae*, *Phikmvvirus*	Podovirus	*P. aeruginosa* CN573	OP292288	42.7	Pili	~27%^*c*^	(20)
PT07	*Caudoviricetes, Pakpunavirus*	Myovirus	*P. aeruginosa* CN573	OQ850183	94.7	MexAB-OprM^*d*^	17%^*c*^	(21)

^*a*^ Staphylococcal Twort-like bacteriophages like ISP are known to have at least two receptor binding proteins: the glycosylated wall teichoic acids (WTAs) and the WTA backbone.

^*b*^ The host range was calculated based on clinical data from 92 *S. aureus* strains, collected between 2020-2023 at the Queen Astrid Military Hospital (unpublished data). Only lysis from within results are included.

^*c*^ The host range was calculated based on clinical data from 343 *P. aeruginosa* strains, collected between 2020-2023 at the Queen Astrid Military Hospital (unpublished data). Only lysis from within results are included.

^*d*^ The receptor for PT07 is not known, but sequence similarity to PAK-P1 like bacteriophages (98.26% identity to bacteriophage PaP1) suggests it might be the *P. aeruginosa* MexAB-OprM multidrug efflux pump.

To ensure deposition of sufficient numbers of active phages at the location of infection for the treatment indications included, and hereby avoid application of subtherapeutic dosages, experiments were performed to determine the stability of magistrally prepared phage solutions, when stored in polycarbonate/polypropylene syringes at 4°C and when used in a device (nasal douche for CRS and a three-lumen catheter for sepsis), prior to administration to patients.

### Magistral phage preparation

The QAMH provided the APIs of ISP, 14-1, PNM, and PT07 in Dulbecco’s phosphate-buffered saline (DPBS) with a titer of 1.3 × 10^10^, 5.1 × 10^9^, 2.0 × 10^9^, and 1.0 × 10^10^ PFU/mL, respectively, and pH ranging between 6.0 and 8.0 according to the conformity reports of Sciensano. The API solutions were transported in glass vials (Fig. 1).

Magistral phage solutions were prepared at the Pharmacy Department at the University Hospitals Leuven. The APIs were aseptically diluted with NaCl 0.9% at a pH of 5.5 and drawn up in syringes (Fig. 1). For CRS, 1 mL phage solutions with a titer of 10^9^ PFU/mL were prepared in 1 mL polycarbonate syringes (BD Plastipak, ref. 309628). In a later stage, as previously described, the phage solution is diluted with 99 mL NaCl 0.75%/sodium bicarbonate 0.25% mixture by the patient to obtain a final titer of 10^7^ PFU/mL. For MSI, 20 mL solutions at 10^7^ PFU/mL were prepared in 20 mL polypropylene syringes (BD Plastipak, ref. 300629). In view of CF/Bx applications, using a vibrating mesh nebulizer, 4 mL solutions with a titer of 10^8^ PFU/mL were prepared in 5 mL polypropylene syringes (BD Plastipak, ref. 309649). Finally, a 50 mL solution with a titer of 10^8^ PFU/mL was prepared and stored in a 50 mL polypropylene syringe (BD Plastipak, ref. 300865) for intravenous use in septic patients. Syringes were stored at 4°C (Fig. 1).

### Bacteriophage enumeration

To determine phage stability over time or after the use of a device, phage titers were measured repeatedly using the double-agar overlay method. With this technique, a lawn of bacteria is formed on a solid agar plate after overnight incubation. Active phage particles are added to the plate using a more liquid, soft agar, which explains the name of the “double” agar method. Afterward, these phages infect and lyse the bacteria, whereby the cloudy bacterial cell suspension in the agar disappears. A clear or translucent zone, called a plaque, is then formed (22). Since a plaque arises from a single phage particle, the total number of plaques can be counted and the titer of the tested solution can be defined as the number of plaques per mL (PFU/mL in short).

First, tenfold serial dilutions were prepared in phage buffer (10 mM Tris, 10 mM MgSO_4_, and 150 mM NaCl, adjusted to pH 7.5). Next, 100 µL of the diluted phage solution was mixed with 200 µL of overnight grown host bacterium (ATCC6538 for ISP and CN573 for PNM, 14-1, and PT07) and 4 mL of lysogeny broth soft agar (0.7%). The mixture was dispersed evenly on solid tryptic soy agar plates (bioTRADING Benelux B.V.). After overnight incubation at 37°C, translucent plaques were counted and the titer (PFU/mL) was calculated, as previously described. Titers of each sample were determined in triplicate.

### Stability of magistral phage preparations in polypropylene/polycarbonate syringes

To assess the stability in polypropylene/polycarbonate syringes, the phage titer was determined repeatedly. For CRS and MSI, the titer was tested on days 1, 2, 5, and 7. For CF/Bx, the experiment was prolonged, based on the treatment protocol, and titers were also assessed on days 10, 12, 14, 16, and 21.

### Mechanical test for CRS and sepsis

For CRS, a mechanical test was performed to determine the impact of squeezing the nasal bottle manually, and thus applying positive pressure. A 1-mL phage solution (10^9^ PFU/mL) was diluted with 99 mL NaCl 0.75%/sodium bicarbonate 0.25% mixture (37°C) in a plastic nasal douche (Nasofree, 250 mL). The titer was assessed at baseline (T_−1_), after squeezing the bottle immediately after dilution (T_0_) and after storing for 60 min at room temperature (T_1_). For sepsis, the stability after infusion through a three-lumen central venous catheter (ARROW, ref. CV-15703) was determined by measuring the titer at baseline (T_0_) and after infusion for 5 min (T_1_).

Finally, the adsorption of phages to the inner wall of polypropylene 50 mL syringes (sepsis) was tested on day 7. Syringes containing the phage solution were completely emptied and the number of active phages (PFU and PFU/mL) at baseline was determined. Afterwards, Tween 20 (0.1%) was added to the syringe, after which it was placed on a shaking platform at 4°C for 45 min. The number of active phages (total PFU, PFU/mL) was calculated. This process was repeated for three cycles. Tween 20 (polysorbate 20) is a nonionic detergent that is widely used as a solubilizing agent, stabilizer, and washing agent. It acts by modifying the integrity and structure of surface and free-floating vesicles, increasing the hydrophilicity of the surface, improving surface passivation, and inhibiting the nonspecific binding between biomolecules and the surface (23). The detergent does not affect phage viability as it is often used as a washing agent, e.g., for phage-tagging in the setting of live-dead stainings (24).

### Statistical analysis

Statistical analysis was performed using GraphPad Prism (version 9.4.1.). For each time point, the titer was assessed in triplicate and mean and standard deviation were calculated. Differences in continuous data, with/without normal distribution, were assessed using a paired *t* test and Wilcoxon test, respectively. Additionally, differences between more than two variables were measured using the one-way analysis of variance (ANOVA), for paired data with normal distribution, or the Friedman test, for paired data without normal distribution. Multiple testing was corrected with the built-in Dunnett test for one-way ANOVA. Normality was determined using the Shapiro-Wilk test. *P* values <0.05 were considered statistically significant and 95% confidence intervals (CIs) were calculated.

### Conclusion

Stability testing is of key importance before human application of magistral phage preparations. A decreased titer after storage of a phage solution can lead to the administration of subtherapeutic concentrations. In our experiments, we demonstrated that phage solutions can be safely stored in polypropylene/polycarbonate syringes at 4°C for a maximum of 7 days. Afterward, the stability of the titer above the predefined ETT cannot be guaranteed. Phage PT07 seemed to be less stable over time, especially in polycarbonate syringes. In addition, phage titers remained stable after rinsing with a nasal douche and irrigation through a catheter.
